# Bacterial vaccines in poultry

**DOI:** 10.1186/s42269-019-0260-1

**Published:** 2020-01-29

**Authors:** Nagwa S. Rabie, Zeinab M. S. Amin Girh

**Affiliations:** Department of Poultry Diseases, National Research Centre, 33 Bohouth St, Dokki, Giza, Egypt

**Keywords:** Poultry, Bacterial vaccines, Live vaccines, Inactivated vaccines, Subunit vaccines, DNA vaccines

## Abstract

**Background:**

Poultry bacterial pathogens are mainly controlled by using high-cost sanitary measures and medical treatment. However, the drug-resistant strains of pathogens continuously emerge, and medical treatments are often ineffective. Moreover, there is increasing public objections to drug residues in poultry products. The other important type of control is the vaccination which depends on immunity. This immunological control is the major practical alternative to chemotherapy. Success of vaccines in combating poultry diseases depends mainly on the choice of the proper type of vaccines, correct time of its usage, and method of administration.

The types of vaccines include attenuated live vaccines, and these vaccines were shown to be effective in inducing protection. The second type is killed vaccine or whole bacteria extracts which is less successful in providing protection compared to live vaccines. The metabolic product vaccine (toxoids) is the third type of vaccine. The recombinant DNA technique was adopted to produce the protective antigens in a sufficient amount and in cost-effective ways.

**Conclusions:**

Protection studies against bacterial diseases were performed by using several trials: living vaccines (live attenuated vaccines; live, non-pathogenic microorganisms; live, low virulence microorganism), inactivated (killed) vaccines (heat-inactivated, chemical inactivates, radiation), metabolic product vaccines (toxoids), subunit vaccines (whole cell proteins, outer membrane proteins, purified flagellar proteins (flagellin), fimbrial proteins, pilus proteins, lipopolysaccharides), vaccines produced by recombinant deoxyribonucleic acid (DNA) technology, and DNA vaccines.

## Introduction

Bacterial infections of poultry are a worldwide important factor in terms of their economic losses and public health. The control of these diseases depends on high-cost sanitary measures and medical treatment. However, the appearance of bacterial strains resistant to these drugs may be due to the overuse of antibiotics as well as due to care about the effect of drugs and their residues on poultry products. Moreover, some organisms are naturally resistant to most antibiotics (Nakae et al. 1997), and all these reasons led to searching for other immunological means of control. One of which was vaccination which was found very effective in providing protection against bacterial diseases. The protection effect of bacterial vaccines depends on the immune response of the host towards different antigenic components of the bacteria. Antigens stimulate humoral immunity (mainly B cells, which give rise to antibodies and cooperate with eosinophils, macrophages and neutrophils) and cell-mediated immunity (mainly T cells, which produce lymphokines). Bursa of Fabricius and the thymus serve as the primary lymphoid organs of the immune system. B cells use surface immunoglobulins as antigen receptors and differentiate into plasma cells to secrete antibodies. Three classes of antibodies are produced: IgM, IgG (also called IgY), and IgA. Successful vaccinal response in a flock is often monitored by demonstrating a rise in antibody titer within a few days of vaccination. ELISA is used most commonly for serologic monitoring. T cells are the principal effector cells of specific cellular immunity. T cells differentiate into alpha beta and gamma delta cells. In adult birds, gamma delta cells may constitute up to 50% of the circulating T cells. Functionally, CD4+ cells serve as helper cells and CD8+ cells as cytotoxic/suppressor cells (Sharma 1999).

The development of bacterial vaccines depends on different techniques, live or inactivated (killed) forms of bacteria. Live attenuated vaccines against several poultry diseases provided protection and were commercially available. Some of these diseases are mycoplasmosis in turkeys and chickens caused by *Mycoplasma gallisepticum* (*M. gallisepticum*) (Ley et al. 1997) or *Mycoplasma synoviae* (*M. synoviae*) (Morrow et al. 1998), fowl typhoid caused by *Salmonella enteritidis (S. entertidis*) (Babu et al. 2003) or *Salmonella gallinarium* (*S. gallinarum*) (Barrow et al. 2000). Paratyphoid caused by *Salmonella typhimurium* (*S. typhimurium*) (Bachtiar et al. 2003), colibacillosis caused by *Escherichia coli* (*E. coli*) (Peighambari and Gyles 1998), and fowl cholera caused by *Pasteurella multocida* (*P. multocida*) (Scott et al. 1999). Some of the disadvantages of live attenuated vaccines are that they are based on living organisms which face problems in preparation (as contamination) and batch uniformity. They provide limited immunity if severely attenuated and may cause diseases if insufficiently attenuated.

Other forms of living vaccines prepared from non- pathogenic microorganisms as in salmonellosis (Hassan and Curtiss 1997), colibacillosis (Frommer et al. 1994), and low virulent microorganisms as in campylobacteriosis caused by *Campylobacter jejuni (C.jejuni)* (Ziprin et al. 2002) gave no protection.

Inactivated (killed) vaccines are prepared from whole bacterial preparation combined with an adjuvant. They are inactivated by either heat at 60 °C for 1 h as in campylobacteriosis (Widders et al. 1998) or chemicals as in samonellosis (Duchatel et al. 1998) and in fowl cholera (Khafagy et al. 1999) or radiation as in *Pseudomonas* infection (Mohamed et al. 2002).

Metabolic product vaccines as toxoids (Fukutome et al. 2001) and subunit vaccines are prepared from outer membrane proteins (Abd-Aty and Rabie 2003) whole-cell proteins and flagellin (Rabie and Zou El Fakar, 2004), fimbrial and pilus proteins, and lipopolysaccharids (Shujian et al. 1996).

Recombinant DNA technology as in salmonellosis (Dueger et al. 2003) and DNA vaccines are used mainly in viral infections.

## Traditional techniques of bacterial vaccine production

Conventional methods of bacterial vaccine development is based on whole bacteria, and they are divided into two groups: living vaccines and inactivated (killed) vaccines.

### Living vaccines

#### Live attenuated vaccines

In this type of vaccine, the living microorganisms are either avirulent or rendered avirulent by attenuation; this means that these pathogens are capable of multiplication within the host but are incapable of causing diseases. Live attenuated bacteria simulate natural infection which increases cell-mediated immune response. Immunization of day-old chicks with the attenuated *S. typhimurium* live vaccine strain resulted in the same change in T cell composition as seen after infection with the non-attenuated salmonella wild-type strain, but at a lower level except an increase of CD_8+_ TCR_1+_ (gamma delta) double-positive cells which have an important role in the immunological defense of chickens against *salmonella* exposure (Berndt and Methner 2001). *S. enteritidis* live attenuated vaccine was more effective in increasing T lymphocyte proliferation than killed vaccine in laying hens (Babu et al. 2003). Vaccination of layer chickens with a live attenuated *S. gallinarum* 9 R strain reduced *S. enterica* infections and the vaccine could not spread to the egg content (Faberwee et al. 2001). The live *Mycoplasma gallisepticum* (*M. gallisepticum*) vaccine strains ts-11 and 6/85 could be transmitted from vaccinated layer pullets to unvaccinated pullets, broiler breeders, turkey breeders, or meat turkeys which were in indirect contact with them (Ley et al. 1997).

##### The pathogenic live bacteria was attenuated by mutagenesis

Chemical mutagenesis of bacteria by using N-methyl N′-nitro-N nitroguanidine (NTG) for production of clones with a temperature sensitive (ts) and used as a vaccine candidate in *Mycoplasma synoviae (M. synoviae)* in chickens (Morrow et al. 1998; Markham et al. 1998) and *Ornithobacterium rhinotracheale* (*O. rhinotracheale*) in turkeys (Lopes et al. 2002). Broiler chicken breeders were vaccinated with a temperature sensitive mutant of *M. gallisepticum*; the vaccine prevented infection in tracheas and infra orbital sinuses of these breeders and in vitelline membrane of their embryos. In addition, the broiler offsping of the vaccinated breeders had better production performance (Barbour et al. 2000).

Intramuscular administration of the aro A-ser C (aromatic dependent mutants) of the lysogenic strain of *S. gallinarium* induced protection against experimental fowl typhoid (Barrow et al. 2000). An aro A-attenuated mutant of *S.* Typhimurium vaccine was used as heterologous antigen delivery and prevent salmonellosis in chickens (Bachtiar et al. 2003). Attenuated live vaccine was prepared from respiratory chain muations (nuoG, 47GyoA, atpB, and at pH) of three *Salmonella enterica* servoars Typhimurium*,* Gallinarum and Dublin in chickens and mice (Turner et al. 2003). Attenuated derivatives (Delta Cya Delta crp mutants) of O2 and O78 avain septisemic *Escherichia coli* (*E. coli*) strain were used to immunized broiler chickens by spraying. The mutant O2 strain provided moderate protection against airsacculitis but not the O78 strain (Peighambari et al. 2002). Live attenuated streptomycin-dependent (str-dependent) mutants derived from a virulent APEC did not cause mortality in challenged birds and systemic lesions were significantly reduced when birds were given three vaccinations on days 1 (aerosol), 14 (oral), and 28 (oral) (Amoako et al. 2004)*.*

Vaccination of fattening turkey flock with live commercial *S. enteritidis* at day 1 of age via spray and boostered at 6 and 11 weeks of age via drinking water did not reduce shedding or colonization of internal organs when birds were challenged with *S. enteritidis* PT4 (Krüger et al. 2008)*.*

#### Live, non-pathogenic microorganisms

A live, non-pathogenic piliated strain of *E. coli* was reported to be effective when broilers were vaccinated by the oral or intramuscular route at 14 or 21 days of age and challenged by the intramuscular route 1 week later with virulent avian pathogenic *E. coli* while vaccination by spraying did not induce adequate protection (Frommer et al. 1994).

Oral immunization with *Haemophilus somnus* vaccine protected broilers from infection with virulent strains of *S. enteritidis* (Wieliczko et al. 2000).

Attenuated or avirulent bacteria can be used as vehicles for the effective delivery of vaccine candidates (Rappuoli et al. 2011). Attenuated *Salmonella* strains are often used in poultry for the control of salmonellosis and they can serve as safe and effective oral carrier vaccines to prevent NE by expressing heterologous antigens (Jiang et al. 2015).

#### Live, low virulence microorganism

Intravenous infection of pigeons with the low virulence *Streptococcus gallolyticus* (*S. gallolyticus*) serotype I strain PDH827 did not induce clinical protection against challenge with high virulence serotype I strain STR357 (Kimpe et al. 2002). The treatment of chicks with viable non-colonizing strains of *C. jejuni* intramuscularly as a possible vaccine with or without adjuvant was failed to induce protective immunity (Ziprin et al. 2002).

### Inactivated (killed) vaccines

Inactivated vaccines are generally whole bacterial preparations combined with an adjuvant and the cultured pathogens are rendered non-infectious by killing and are used for immunization. Killed bacterial vaccine are called bacterin, and killing microbial pathogens is achieved by using one of the following methods:

#### Heat inactivated (60 °C for 1 h)

Vaccination of broilers and layers with Tribacto-pulvis heat inactivated vaccine which was prepared from *Salmonella*, *E. coli*, and *P. aeruginosa* resulted in reduction in death by 30–50%, increasing in weights 100–200 g and less coccidiosis and infectious bronchitis in vaccinated birds (Cambir 1999). Chick embryos were orally immunized at day 16 of incubation by injection of heat-killed *C. jejuni* organisms into the amniotic fluid which increased antibody response in hatched chicks (Noor et al. 1995) which was higher than that of embryos immunized with soluble *C. jejuni* antigen (Noor 1998). Effective inactivated *E.coli* vaccines against serotypes including O2:Kl and O78:K80 have been produced and provide protection against the homologous serogroups not against heterologous serogroups (Saif et al. 2003).

#### Chemical inactivants

They are protein denaturants as formaldehyde, acetone, and alcohol or alkalyating agents as ethylene oxide, ethyleneimine acetylmethylimine, and β-propriolactone. Chickens were vaccinated with formalized antigen of *C. jejuni* with or without immunodulator reduced reisolation of *C. jejuni* from internal organs but did not protect chicks (Rabie and Kutkat 2002). Pigeons were vaccinated with killed vaccines containing whole cell formaldehyde-inactivated *S. typhimurium* Var. Copenhagen. The vaccine could not induce protection against challenge but only reduced fecal shedding (Vereechen et al. 2000). Vaccination of rabbits with a saponin killed vaccine of *Mycoplasma mycoids* resulted in increased humoral immune response (Sunder et al. 2001). Inactivated *S. enteritidis* phagotype four vaccines (emulsified in light mineral oils or adsorbed in aluminum hydroxide) were used in 1-day-old chicks; no reisolation of *S. enteritidis* from cloacal swabs could be abtained after challenge. The vaccines emulsified in mineral oils produced greater antibodies compared to that prepared by adsorption into aluminum hydroxide (Fernchini et al. 1997). Autogenous in activated tissue vaccine (from the liver and spleen of diseased chickens) to be an approach to the prevention of ascites syndrome in broilers in which four bacterial strains were isolated and the *E. coli* was the most commonly isolated strain (Shuxia et al. 1999). Avian *Salmonella* oil vaccine was used in day-old Japanese quail by S/C and I/M injection. No deaths were observed and weak immunological reaction was detected. The egg production was lower only during the period of 6–10 weeks after vaccination (Ito et al. 2000). The comparative efficacy of oil-based and gel-based vaccine adjuvants has been studied by other researchers. Some studies demonstrated that an oil-based vaccine induced a higher antibody level and provided better protection against field strains (Jacobs et al. 1992; Fukanoki et al. 2000; Chukiatsiri et al. 2010; Gong et al. 2014).

#### Radiation

Immunization of chicks with gamma irradiated (cobalt 60) bivalent *Pseudomonoas aeruginosa* (*Ps. aeruginosa*) vaccine recorded protection by 100%, 96%, and 90% post challenge intramuscular, subcutaneously and orally vaccinated chicken groups, respectively; also, the vaccination of layers with the same vaccine stimulated the formation and concentration of *P. aeruginosa*-specific Igy in the egg yolk (Mohamed et al. 2002). Chickens were immunized intraocular with liposome associated *S. enteritidis* antigen; the antigen was prepared by ultrasonicated whole cell extract of the bacteria. The vaccine induced increase in the specific antibody producing lymphocytes in the intestinal tract and immunoglobulin secreted in the intestine inhibited the adherence of the bacteria to intestinal epithelial cells suppressing the spread of the bacterial infection in the host (Fukutome et al. 2001).

##### Combined bacterins

Two mixed bacterins from *E. coli*, *Staphylococcus aureus*, and *Clostridium perifringens* (*C. perifringens*) or *Clostridium septicum* (*C. septicum*) were used for immunization of chickens against gangrenous dermatitis; the vaccines were found to be safe and they protected the birds against challenge with live cultures of the bacteria without any untoward reactions (Kaul et al. 2001)

Both the heat and formalin inactivated aluminum precipitated vaccines prepared with the virulent *E. coli* isolates was effective to protect chickens of different age against various forms of avian colibacillosis (Rashid et al. 2001). A developed combined vaccine was prepared from trivalent *E. coli* vaccine (serotypes O1, O2, and O78) and an inactivated Newcastle disease vaccine induced a high degree of protection in layers and chicks (Erganis et al. 2002).

##### Simultaneous use of inactivated and live vaccines

The use of a live *P. multocida* vaccine followed by a killed *P. multocida* vaccine, two live vaccines, or a killed vaccine followed by live vaccine provides almost equal immunity when measured by enzyme-linked immunosorbent assay (ELISA) titers (Hofacre et al. 1987). Parent chickens were vaccinated with live *S. typhimurium* and inactivated *S. enteritidis* induced an increase in antibody concentration in sera and jejunum of the chicks (Mathner et al. 2002). Laying hens should be vaccinated with live and killed vaccines to stimulate mucosal and systemic immunity and reduce the prevalence of *S. enteritidis*-contaminated eggs (Davies and Breslin 2004)*.*

## Metabolic product vaccines (toxoids)

These are soluble toxins that are rendered harmful (non-toxin) by addition of formalin or by gentle heating; this way does not affect the immunogenic properties of the toxin. Ducks were immunized with a type of botulinum toxin; it induced partial protection but in a single dose while double dose vaccine increased signs of botulism and the vaccine can be used to wild birds during botulism epizootics (Rock et al. 2000). Samonella toxins (enterotoxin plus cytotoxins) were the main virulent products of the organisms formalized (FT) and carbonated (CT) toxoids were prepared from partially purified toxins of *S. enterica* ser. weltevreden and Gallinarum. Complete protection could be obtained in birds vaccinated with FT of *S. weltevreden* plus Freund’s complete adjuvant (FCA) following homologous or heterologous (*S. gallinarum* and *S. typhimurium*) challenges while protection ranged from 50 to 83.3% in the groups immunized with other preparations of *S. weltevreden*, i.e., with FT without FCA or with CT with or without FCA. Gallinarum toxins (FT) given with FCA afforded 100% protection against homologous challenge, but not against heterologous serovars (Mishra and Sharma 2001). Chicks received vit. E adjuvant salmonella toxoid; high lymphocyte stimulation was recorded and the vaccinated chicks were protected against *Salmonella* challenge (Barman et al. 2000). Several trials have shown that chickens could be protected against *C. perfringens-*induced necrotic enteritis (NE) by injection with inactive and active toxins (Jang et al. 2012; Kulkarni et al. 2007) and antigenic proteins (Jiang et al. 2015).

## Subunit vaccines

They are prepared from one or few immunogenic epitopes that are found an infectious agent. Among the surface epitopes of an entigen molecule, few epitopes are important in stimulating protective immunity.

### Whole-cell proteins

Immunization of chickens with surface antigens proteins of *E. coli* induced highly systemic and mucosal antibody responses (Kariyawasam et al. 2002). Ammonum sulfate perceptible protein (ASPP) of *Pasteurella multocida* serotype 6 B yielded three protein fractions, which can be used to develop a subunit vaccine against haemorrhagic septicemia in rabbits (Srivastava 1999). *S*. *enteritidis* OMPs of 75.6 and 82.3 KDa were effective in reducing colonization of *S. enteritidis* on intestinal mucosa in chickens (Khan et al. 2003). Chicks were immunized with *C. jejuni* OMPs vaccine (44–80 KDa); it reduced the infection after challenge and increased the serum antibody titer (Abd-Aty and Rabie 2003).

### Outer membrane proteins (OMPs)

Forty-five kilodaltons protein is considered to be a major OMP of *C. jejuni* and has immunogenic effect in chickens (Lam 1992). The immunodominant protein antigen of *C. jejuni* is subunit molecular weight of 59 to 61 KDa (Dubreuil et al. 1990). Chicks were immunized with *C. jejuni* OMPs vaccine (44–80 KDa); it reduced the infection after challenge and increased the serum antibody titer (Abd-Aty and Rabie 2003). *S. enteritidis* OMPs of 75.6 and 82.3 KDa were effective in reducing colonization of *S*. *enteritidis* on intestinal mucosa in chickens (Khan et al. 2003).

### Purified flagellar proteins (flagellin)

Immunization of broiler chicks with purified native flagellin or combined heat killed *C. jejuni* and flagellin induced reduction in the number of *Campylobacter* in caecal contents. Flagellin (61–63 KDa) and possibly the 67 KDa antigen may be valuable for immunological control of *C. jejuni* and used as vaccine candidates. (Widders et al. 1998). Flagella and whole cell extraction were used as antigens for detection of antibodies to *S. enteritidis* in serum and egg yolk of infected hens by agar gel preciptin test while SEF14 (a 14-KDa fimbrial protein) was not reactive (Holt et al. 2000). Immunization of chicks with *P. aeruginosa* whole cell proteins of strain D and E (20–205 KDa for each strain) and flagellin (53.277 KDa and 54.184 KDa, respectively) revealed high immunological responses and reduced infection in chicks but the whole cell oil adjuvant vaccine recorded best results than the flagellar oil adjuvant vaccine (Rabie and Zou El Fakar 2004).

### Fimbrial proteins

Immunization of hens with Sef A and Fim A fimbrial proteins of *S. enteritidis* induced strong humoral immune response similar to that obtained with live bacteria. Sef A and Fim A can be considered as components of subunit vaccines (Kisiela et al. 2003)

### Pilus proteins

Intranasal vaccination of broiler chickens with four avian pathogenic *E. coli* surface antigens, F pilus adhesin, P pilus adhesion, aerobactin receptor protein, and lipopolysaccharide (LPS) induced high immune response (high titer of IgG, IgA, and IgM) and did not induce the disease after challenge. They appear to be suitable candidates for a vaccine (Kariyawasam et al. 2002).

### Lipopolysaccharides

Capsular polysaccharide subunit vaccines for *E. coli* can be prepared by extraction of capsular polysaccharide and soluble bacterial protein through water–bath inactivation and used for immunization of chickens and provided high protection (Shujian et al. 1996).

## Vaccines produced by recombinant DNA technology

These vaccines depend upon identification or isolation of antigenic-coding gene. Then using recombinant technology transgenic implementation of the isolated gene in a bacterial vector like *E. coli* or yeast cells is performed. The expressed gene products of the grown culture is purified and used for immunization. Messenger RNA, which codes for the chosen proteins, is copied to produce a complementary DNA (cDNA) strand. This DNA strand can also be copied to produce a second strand. The double-stranded form of cDNA is then ligated to a cloning vector (plasmid) which is ready to be *cloned* using cloning host. The cloned recombinant DNA (rDNA) is then expressed by transformation into bacterial vector usually *E. coli* or yeast cells which act as production factories for the selected protein. The produced recombinant antigen is identified using selectable markers. These identified recombinant proteins could be injected into birds or animals as vaccine candidate (Babiuk et al. 2003). The immunization of chickens with a temperature-sensitive mutant E/1/3 of *S. enteritidis* induced strong protection against virulent *S. entritidis* strain after oral challenge and reduced the caecal and spleen colonization and the number of faecal shedding. (Cerquetti and Gheradi 2000). Salmonella DNA adenine methylase mutants prevent colonization of newly hatched chickens by homologous and heterologous serovars (Dueger et al. 2003). Gene E leads to emptying pasterulla cell envelops which are called bacterial ghosts*. P. multocido* and *Pasteurella haemolytica* (*P. haemolytica*) ghosts produced by expression of phage phi X174 lysis gene E are used as a vaccine for immunization of rabbits producing 100% protection (Marchart et al. 2003). Three *Campylobacter jejuni* 72/D2/92 genes (CjaA (omph), cjac (hisj), and cjaD (omp18)) encoding immunodominant proteins are considered to be potential chicken vaccine candidates (Pawelec et al. 2000).

## DNA vaccines

DNA encoding the gene of antigen protein is ligated to a plasmid. Direct inoculation of this plasmid DNA into the host tissues which is able to cause expression of the encoded antigen protein within the transfected cells. The expressed protein stimulates the host immune system to produce specific immune responses. DNA vaccine offers many advantages over the previously mentioned vaccines; there is no risk of infection, no purification costs, or antigen denaturation during preparation. Also, the endogenous synthesis of microbial antigen strongly enhances the cell mediated immunity; its strong stability reduces the costs of cold chains requirement by 80%. DNA vaccines do not interfere with the maternal immunity and single dose can induce long term immunity (Oshop et al. 2002).

## Conclusion

Protection studies against bacterial diseases were performed by using several trials: living vaccines (live attenuated vaccines; live, non-pathogenic microorganisms; live, low virulence microorganism)

Inactivated vaccines (heat-inactivated, chemical inactivates, radiation) are metabolic product vaccines (Toxoids), subunit vaccines (whole cell proteins, outer membrane proteins, purified flageller proteins (flagellin), fimbrial proteins, pilus proteins, lipopolysaccharides), vaccines produced by recombinant DNA technology, and DNA vaccines. The chicken farms must be care for using bacterial vaccines.

## Recommendations

Bacterial vaccines need more investigations and researches because most farms depend on the use antibiotics for treatment when spread of bacterial diseases.

## Data Availability

All data collected in this study are included in this published article.

## Abbreviations

*C. jejuni*: *Campylobacter jejuni*
*E. coli*: *Escherichia coli*
*M. gallisepticum*: *Mycoplasma gallisepticum*
*M*. *synoviae*: *Mycoplasma synoviae*
NTG: Nitroguanidine
*O. rhinotracheale*: *Ornithobacterium rhinotracheale*
*P. multocida*: *Pasteurella multocida*
*Ps. aeruginos*: *Pseudomonoas aeruginosa*
*S. enterica*: *Salmonella enterica*
*S. entertidis*: *Salmonella enteritidis*
*S. gallinarum*: *Salmonella gallinarium*
*S. gallolyticus*: *Streptococcus gallolyticus*
*S. typhimurium*: *Salmonella typhimurium*
